# MiR-375: it could be a general biomarker of metabolic changes and inflammation in type 1 diabetes patients and their siblings

**DOI:** 10.1007/s40618-024-02474-4

**Published:** 2024-10-25

**Authors:** Eman A. Mostafa, Nagwa Abdallah Ismail, Abeer M. Nour El Din Abd El Baky, Tarek F. ElShaer, Ingy Ashmawy, Aliaa Ahmed Wahby, Mai Magdy Abdel Wahed, Shereen Hamdy Abd El Aziz

**Affiliations:** 1 National Research Center, Department of Pediatrics, El Buhouth St., P. O. 12622, Dokki, Cairo, Egypt; 2 National Research Center, Department of Clinical and Chemical Pathology, Cairo, Egypt

**Keywords:** T1D: Type 1 diabetes, RT-PCR: Real-time polymerase chain reaction, MiRNAs: MicroRNAs, HbA1c: Glycated hemoglobin A1c, GADA: Glutamic acid decarboxylase autoantibodies, ICA: Islet cell autoantibodies

## Abstract

**Purpose:**

Type 1 diabetes (T1D) is a chronic autoimmune illness that results in loss of pancreatic beta cells and insulin insufficiency. MicroRNAs (miRNAs) are linked to immune system functions contributing to the pathophysiology of T1D, miRNA-375 is significantly expressed in the human pancreas and its circulatory levels might correspond to beta cell alterations. Pancreatic islet cell antibodies (ICA) and Glutamic acid decarboxylase antibodies (GADA) have roles in autoimmune pathogenesis and are predictive markers of T1D. The aim of this work was to detect serum level changes of miRNA-375, ICA, and GADA in T1D patients, and their siblings compared to healthy controls and correlate them with T1D biochemical parameters.

**Methods:**

The study included 66 T1D patients (32 males and 34 females; age range 3–18 years), 22 patients’ siblings (13 males and 9 females; age range 4–17 years), and 23 healthy controls (7 males and 16 females; age range 4–17 years). MiRNA-375 levels were measured using quantitative reverse transcription polymerase chain reaction (RT-qPCR), while ICA and GADA levels were measured using enzyme-linked immunosorbent assay (ELISA). Data analysis was done utilizing SPSS-17 software.

**Results:**

MiR-375 levels were downregulated in T1D patients and further decreased in their siblings when compared to healthy controls. Furthermore, miR-375 exhibited inverse correlations with HbA1c levels but no correlations with Total Insulin Dose, disease duration, or autoantibodies (GADA & ICA).

**Conclusion:**

Our study indicates that miR-375 is significantly downregulated in children with T1D and their siblings, suggesting its potential role as a biomarker for beta-cell function and glycemic control.

**Supplementary Information:**

The online version contains supplementary material available at 10.1007/s40618-024-02474-4.

## Introduction

Anti-islet autoantibodies are important markers for immune-mediated type 1 diabetes T1D and slow-progressing T1D. Autoantibodies against tyrosine phosphatase-like protein IA-2 (IA-2 A), glutamic acid decarboxylase (GADA), insulin (IAA), and zinc transporter 8 (ZnT8A) are being used in the diagnosis, pathological examination, and identification of (T1D) [1].

Growing evidence reveals that microRNAs (miRNAs) have a vital role in the function of the immune system as well as beta-cell metabolism, proliferation, and death, all of which are included in the pathophysiology of T1D [2].

MiRNAs are tiny non-coding RNAs composed of 19–22 nucleotides that serve as negative regulators of the expression of genes by partially pairing with the 3’ or 5’ untranslated regions of their target mRNAs. Several investigations found that miRNAs can be recognized in extracellular fluids and body secretions as well [3].

Circulating miRNA levels are being proposed as a novel indicator for the detection and prognosis of various disorders, such as T1D, as well as potential targets for therapy and intervention [4].

*Mir 375* has abundant expression within the glandular pancreas and was initially derived from cell lines secreting insulin. In vitro investigations revealed that Mir 375 has a role in the islet’s cell formation, suppresses the expression of insulin genes when exposed to glucose [3], and influences voltage-gated sodium channels [5].

Whereas dysregulated *miRNA* profiling has been noticed in T1D patients, *miR-375* is considered the most potential biomarker for beta cell death [6]. However, results in T1D patients are mostly inconsistent, with some research revealing increased circulating miR-375 levels, while others show unchanged or lower levels when compared to those without diabetes [4].

Higher levels of miR-375 in people with T1D imply autoimmune activity or damage to remaining β-cells and cessation of insulin release [7]. Meanwhile, Marchand et al. (2016) discovered that miR-375 was lower in T1D kids compared to their age-matched peers.

Previous studies on miR-375 were done in small groups of affected individuals and in different types of specimens such as body fluids, cultured cells, and solid tissue derived from patients with T1D [3, 4], resulting in conflicting findings. Research in large homogenous groups is needed to assess if miR 375 concentrations are altered in T1D patients and whether miR 375 is a possible predictor of beta cell damage.

Egypt has the highest estimated number of childhood-onset T1D cases among Eastern Mediterranean and Middle Eastern nations, accounting for almost 25% of the total [8]. Genetics play an important role in the onset of T1D with strong familial aggregation siblings have an average risk prevalence of 6%, although the general population’s risk is just 0.6% [9]. Moreover, first years following T1D diagnosis, the incidence of the disease onset among siblings can increase up to 5.8% [10].

We hypothesized that miR-375’s expression influences insulin production and beta cell mass in type 1 diabetes, which in turn furnishes its circulating levels as an early indicator of disease development and progression. So, we aimed to assess serum level changes of miRNA-375, ICA, and GADA to Correlate them with different biochemical parameters in a selected number of Egyptian children, including T1D patients, their siblings, and healthy controls.

## Materials and methods

This case-control study consisted of 111 children divided into 66 T1D patients, 22 siblings, and 23 healthy control participants. All participating patients were diagnosed with T1D, collected from our Pediatrics Endocrine Clinic in the Centre of Excellence in the National Research Centre. Inclusion criteria all patients that participated were already clinically diagnosed with T1D by a physician according to WHO criteria such as hyperglycemia, insulin requirement from diagnosis, recurrent infections, high levels of glycosuria, increased urine volume, and thirst, unexplained weight loss, severe cases coma and drowsiness. Diabetic patients were receiving no treatments except insulin at the time when their blood samples were being obtained for this study. Exclusion criteria included children with secondary DM, children with T2DM, and a history of liver or kidney disease and malignancy.

We obtained informed consent from the children’s parents following the criteria of the NRC’s ethical committee in Dokki, Egypt, after the research protocols were carefully explained to them and agreed to share clinical data, blood sampling, and their rights to discontinue at any time. The research protocol was approved by the medical ethics committee of the National Research Centre, Cairo, Egypt, NO;19,207.

### Every research group underwent

The patient’s age, gender, the type and dose of insulin therapy, the start and duration of diabetes, and family history of diabetes should all be included in the history. Any symptoms that could indicate neurological, cardiac, renal, or autonomic illness. Clinical examination including general and local exams. All of the participants underwent blood pressure measurement along with auxological assessment, which included height, weight, hip circumference, waist circumference, and waist-to-hip ratio (cm/cm). *BMI* was estimated by dividing weight by height square (kg/m2), as periodic monitoring of growth parameters is the most significant tool accessible to a pediatric diabetologist for providing current knowledge on linear growth in kids and teenagers with *T1D*.

The HbA1C goal for T1D across all pediatric age groups, of 7.5% (58mmol/mol) is recommended [11].

Each patient performed self-monitoring (or by parents) of blood glucose at seven points a day to assess glycemic targets.

### Sampling

Following a 12-hour overnight fast, all participants had their venous blood drawn. Blood samples for glycated hemoglobin (HbA1C) and complete blood count *(CBC*) assessment were withdrawn into an Ethylene-diamine tetra-acetic acid (EDTA) tube, while the remainder of the sample was transferred to a plain vacutainer tube, which was left to clot and serum was separated.

Part of sera was used for assessment of lipid profile (serum total cholesterol, triglycerides, HDL, and LDL) and liver enzymes (aspartate aminotransferase [AST] & alanine aminotransferase [ALT]).

The other part was used for measuring serum levels of ICA and GADA by enzyme-linked immunosorbent assay (ELISA) and determining serum miR-375 expression using the quantitative reverse transcriptase - polymerase chain reaction (QRT- PCR) technique.

### Routine laboratory tests

We used the Sysmex xs-500i Haematology Analyzer (Sysmex, Kobe, Japan) for *CBC* analysis and the Cobas C311 Chemistry Analyzer (Roche Diagnostics International AG, Rotkreuz, Switzerland) for other routine labs such as lipid profile, liver enzymes, and HbA1c.

### Measuring ICA and GADA serum concentrations

Serum samples held at -80 °C were promptly thawed and centrifuged before analysis. ICA was measured using the Human ICA ELISA kit (Cat. No. E-EL-H5416, Elabscience, Florida, USA), which has a detection range of 0.16 ng/ml to 10 ng/ml and a sensitivity of 0.1 ng/ml. GADA was measured using the Human GADA ELISA kit (Cat. No. E-EL-H2395, Elabscience, Florida, USA), which has a detection range of 1.56 ng/ml to 100 ng/ml and a sensitivity of 0.94 ng/ml. Both kits have low coefficients of variation (< 10%), no cross-reactivity or interaction with other analogs, and use the sandwich-*ELISA* approach. The Stat Fax 2100 Microplate ELISA Reader (Florida, United States) was used to get the data.

### Determination of serum miR-375 expression

The miRNA was extracted using the miRNeasy Mini Kit (cat. no. 217004, Qiagen, Hilden, Germany) according to the manufacturer’s instructions, and the quality and quantity of extracted miRNa were determined using the NanoDrop 2000 spectrophotometer instrument (Thermo Fisher Scientific, Massachusetts, USA). Following that, 10 ng of total miRNA was converted to complementary DNA (cDNA) using the miScript II RT Kit (cat. No. 218161 Qiagen, Hilden, Germany) and the T-100 thermal cycler (Bio-Rad, California, USA). *Q-PCR* was used for miRNA expression profiling with the miScript SYBR Green PCR Kit (cat. No. 218073 Qiagen, Hilden, Germany) and specific miRNA primers (cat. No. 218300 Qiagen, Hilden, Germany) for miR-375 and RNU6 (housekeeping gene). The reaction mix was made for each primer in each sample as follows: Add 12.5 µl SYBR Green PCR Master Mix, 2.5 µl of miScript Universal Primer, 2.5 µl of primer assay, 2.5 µl of RNase-free water, and 5 µl of diluted Template cDNA (1 ng cDNA per well). The Stratagene MX3000P Q-PCR System (Agilent, California, USA) was used to perform Q-PCR. The protocol included an initial activation phase at 95ºC for fifteen minutes, this was followed by forty cycles of denaturing (15 s at 94ºC), annealing (30 s at 55ºC), and expansion (30 s at 70ºC). Florescence collecting information occurs throughout the extension process. The melting curves were used to confirm the validity of PCR results. All devices used are regularly calibrated and randomly selected 10% of samples were duplicated to ensure reliability and accuracy of the results. Data were analyzed using the 2-∆∆Ct technique [12].

### Statistical analysis

The data analysis was carried out utilizing commercialized SPSS software (SPSS, a 17.0; IBM Inc., Chicago, Illinois, USA). Descriptive characteristics of qualitative variables were provided using frequency and percentage. Results for continuous variables were presented as mean ± SD. The normally distributed quantitative variables were examined using the ANOVA test, as well as the post-HOC and LSD tests. Pearson and Spearman’s correlations were used to investigate associations between normal and non-normal continuous variables. Correlations between miR375 levels with clinical and biochemical parameters were calculated A receiver operating characteristic (ROC) curve analysis was conducted to examine the predictive usefulness of ICA islet cell and GADA glutamic acid decarboxylase autoantibodies, as well as mir 375, for T1D diagnosis. Chi-square tests were performed to compare categorical parameters across the study groups. A p-value of < 0.05 was regarded to be statistically significant.

## Results

### The descriptive clinical and laboratory data of the research groups

The study encompassed111 participants divided into three groups: 66 patients with T1D (32 males and 34 females; mean age 12.32 ± 3.48years), 22 siblings of patients (13 males and 9 females; mean age 9.60 ± 3.99years), and 23 healthy controls (7 males and 16 females; mean age 10.73 ± 3.34years). All patients with diabetes were on an intense insulin treatment regimen.

T1D patients and their siblings demonstrated a significant correlation with *SBP* (*p* < 0.001), *DBP* (*p* = 0.033), WC (*p* < 0.001*)*,* HbA1c* (*p* < 0.001), *HDL* (*p* = 0.002), *LDL* (*p* = 0.002), *ICA* (*p* = 0.001), and *GADA* (*p* = 0.043) when compared to control peers as shown in Table 1.

**Table 1:** The desriptive clinical and laboratory data of the research groups

Parameter	Group one n = 66 Mean ± SD	Group two n = 22 Mean ± SD	Group three n = 23 Mean ± SD	ANOVA Significance
Age (years)	12.32 ± 3.48	9.60 ± 3.99	10.73 ± 3.34	0.006
Males No.(%) Females No.(%)	32(48.5%) 34(51.5%)	13(59.1%) 9(40.9%)	7(30.4%) 16(69.6%)	0.134
BMI (Kg/m^2^)	20.51 ± 4.36	19.49 ± 6.27	19.03 ± 2.83	0.347
SBP (mmHg)	106.52 ± 11.96	64.41 ± 12.46	106.74 ± 9.49	0.000^*^
DBP (mmHg)	69.09 ± 8.36	75.36 ± 14.76	70.22 ± 6.48	0.033^*^
W.C (cm)	72.18 ± 12.65	97.27 ± 10.43	66.35 ± 9.31	0.000^*^
W/H ratio	0.87 ± 0.08	0.86 ± 0.05	0.85 ± 0.03	0.299
HbA1c %	8.05 ± 1.59	4.74 ± 0.53	4.67 ± 0.58	0.000^*^
T.Cholesterol (mg/dl)	176.29 ± 32.16	162.91 ± 24.33	176.35 ± 31.73	0.413
Triglycerides (mg/dl)	87.18 ± 37.01	73.09 ± 14.66	77.26 ± 32.92	0.295
HDL-c (mg/dl)	53.90 ± 14.49	47.09 ± 7.97	62.78 ± 9.77	0.002^*^
LDL-c (mg/dl)	82.92 ± 29.30	60.55 ± 12.21	98.04 ± 28.62	0.002*
ICA (ng/ml)	0.643 ± 0.506	0.210 ± 0.091	0.098 ± 0.056	0.001^*^
GADA (ng/ml)	74.74 ± 67.89	113.39 ± 70.66	73.87 ± 41.53	0.043^*^

Group 1 = T1D patients, Group 2 = siblings, Group 3 = healthy controls

Data are presented as mean ± SD, P^*^ < 0.05 is significant, BMI: body mass index, SBP: systolic blood pressure, DBP: diastolic blood pressure, W.C: waist circumference, W/H: waist to hip ratio, HbA1c: glycosylated hemoglobin, Hb: hemoglobin, MCV: mean corpuscular volume, HDL-c: high-density lipoprotein cholesterol, LDL-c: low-density lipoprotein cholesterol, ICA: islet cell autoantibodies, GADA: glutamic acid decarboxylase autoantibodies

### MiR-375 status in the research groups

Table 2 showed statistically significant difference in the levels of miR-375 between three studied groups as P value = 0.000 (1 = T1D 2 = sibling of pt. 1 = controls).

**Table 2:** Mir-375 status in the research group

				95% Confidence Interval For Mean				ANOVA Significance
Parameter	Group	Mean	Standard Deviation	Upper Bound	Lower Bound	Minimum	Maximum	
miRNA 375	1	0.301	0.792	0.107	0.496	0.000	5.389	0.000^*^
	2	0.652	0.875	0.264	1.039	0.016	3.400	
	3	1.181	0.653	0.898	1.462	0.285	2.445	
	Total	0.553	0.851	0.393	0.713	0.000	5.389	

LSD post hoc test results showed that difference in the levels of miR-375 between groups had a significantly lower level of miR-375 in the Diabetic group than in controls (*P* = 0.000). The controls had a significantly higher level than cases and Sibling(*P* = 0.000&*P* = 0.25).

### Correlation of miR-375 with clinical and biochemical parameters in the studied groups

Table 3 showed that miR-375 had a high significant positive correlation with LDL-c, HDL-c, MCV, and AST and a significant negative correlation with HbA1c, without any correlations with total insulin dosage, disease duration, sum of complication, BMI, WC, W/H ratio and autoantibodies (GADA & ICA).

**Receiver operating characteristic (*ROC*) curve analysis was used to determine the predictive value of*ICA*islet cell autoantibodies**, ***GADA*glutamic acid decarboxylase autoantibodies**,** and mir 375 on diagnosis of**T1D.

**Table 3:** Correlation of mir-375 with clinical and biochemical parameters in the studied groups

		mir-375	LDL-c	HDL-c	MCV	AST	HbA1C
mir-375	Pearson Correlation	1	0.387^**^	0.328^**^	0.272^**^	0.390^**^	0.367^**^
	Significant (2-tailed)		0.000	0.001	0.007	0.001	0.000

^*^ Is a significant correlation at 0.05 level (2-tailed)

^**^ Is a significant correlation at 0.01 level (2-tailed)

We constructed (a *ROC*) curve to analyze the potential role of miR 375 levels in predicting T1D in Egyptian children, as shown in Fig. 1 present as a supplementary file.

Table 4 illustrated that AUC (area under the curve) for ICA was superior to those for miR375 and GADA for the prediction of T1D. The AUC was 0.761 (0.671–0.850) for ICA, 0.158 (0.081–0.234) for miR 375 and 0.394 (0.289–0.499) for GADA. The (AUC) performs a good general measure of predictive accuracy. ICA ng/ml had the highest AUC 0.761, so it was the best predictive of T1D. As regards, miR375 AUC was 158, but *p* = 0.000 means the test could discriminate between T1D cases and normal children. When a larger sample is used better AUC could be detected.

**Table 4:** Area under the Curve for miR-375, ICA ng/ml, and GADA ng/ml

Test Result Variables	Area Under Curve	Standard Error (a)	Asymptotic Sig (b)	Asymptotic 95% Confidence Interval	
				Lower Bound	Upper Bound
miR-375	0.158	0.039	0.000	0.081	0.234
ICA ng/dl	0.761	0.046	0.000	0.671	0.850
GADA ng/dl	0.394	0.053	0.060	0.289	0.499

ICA: islet cell autoantibodies, GADA: glutamic acid decarboxylase autoantibodies

The test result variable(s): mir 375, ICA ng/ml, GADA ng/ml There is at least one tie between the positive and negative real state groups. Data may be biased

a: Using the nonparametric assumption

b: Null hypothesis: real area equals 0.5

## Discussion

T1D is a chronic autoimmune illness that results in severe loss of pancreatic beta cells and insulin insufficiency [13]. T1D is responsible for about 90% of diabetes incidences in children and adolescents [14]. In Egypt, 1.5% of 1,000 people under the age of 20 were recently diagnosed with T1D [15].

Individuals with an elevated risk of having T1D can be identified using genetic and immunological markers. Autoantibodies to islet cells, such as ICA (islet cell autoantibodies) and GADA (glutamic acid decarboxylase autoantibodies), are predictive indicators in natural history investigations, appearing months to years before diagnosis [16].

Nevertheless, the presence of these autoantibodies does not always result in a decrease in beta cell mass because positive individuals do not always have T1D [17]. As a result, additional biomarkers are required to predict and prevent illness in its early phases.

In T1D, miRNAs influence essential immune system functions in various cells, such as regulatory T-cell lymphocytes [18].

MiR-375 is a substance present in large quantities within pancreatic islets. It functions by decreasing the amount of insulin secreted in response to glucose by focusing on Pdk1 kinase, in addition to genes that are important or related to the release of insulin from cells, including Mtpu, Aifm1, YWHAZ, and Gephyrin (GPHN) so it controls the secretion of insulin [19], pancreatic development, and differentiation of beta cells [20].

We studied the serum levels of miR-375 in T1D patients and siblings to see if it could be a biomarker for β-cell damage, metabolic changes, and inflammation related to the disease.

In our findings, positive family history represented 82%. This is regarded as one of the most important features of TID. T1D shows high familial clustering; the possibility of having T1D is 8–15 times higher in first-degree relatives and twice as high in second-degree relatives [21].

A comparison of healthy control, type 1 diabetic patients and their siblings showed a significant association with *SBP* (*p* < 0.001) and *DBP* (*p* = 0.033), which coincides with Mallory et al. results, indicating that hypertension in children with T1D is likely more prevalent than previously thought. As a result, we advised children with T1D to have more frequent hypertension screenings [22].

High HbA1c levels indicate a lack of good glycemic control in the diabetic children in our study. Glycated hemoglobin levels were found to be significantly linked with T1D. These results emphasize the significance of glycemic control throughout the diagnosis and treatment of *T1D* [23].

We discovered that *miR 375* levels were low in kids with *T1D* and further reduced in their siblings when compared to healthy controls. These findings indicate that circulatory *miR 375* values could suggest the number of functional B cells facing immunological attack; consequently, the nearer the diagnosis, the lower the *miR 375* values are. Given that *miR 375* affects the production of insulin and the mass of beta cells, we hypothesized that its expression in B cells would change in conjunction with its circulating level [3]. The presence of *miR 375* in a large amount in the *miRNA* profile of islet cells revealed its crucial role in pancreatic beta cells in humans [24].

These results are consistent with those of Raza et al., 2023 [23] and Marchand et al., 2016 [3]. Meanwhile, our findings contradict Bertoccini et al., 2019 [25], who found significantly higher levels of miR-375 in first-degree relatives compared with T1D individuals and healthy controls, indicating a higher β-cell residual function.

Further analysis revealed a significant inverse correlation between *miR-375* and glycated hemoglobin values in the examined groups. The negative coefficients of correlation (*r* = -0.367 and *p* = 0.000, respectively) showed that as *miR-375* expression values decline, *HbA1c* values increase. *HbA1c* is an important marker for long-term control of glycemic index, and elevated levels are associated with inadequate glucose management. The discovered inverse correlation showed that *miR-375* might have a role in controlling glucose metabolism and *HbA1c* levels in people with *T1D* or at risk of developing it [23].

Meanwhile, *Latreille et al. (2015)* [26] discovered that *T1D and T2D* patients with good glycemic control have higher circulating levels of miR-375, which could be attributed to the elimination of high glucose concentrations, which downregulated miR-375.

In the context of hyperglycemia and inflammation in T1D, environmental factors such as viruses may alter miR-375 expression [3]. This is consistent with the current awareness regarding illness pathophysiology, including the potential participation of viral infections [27].

Our study’s ROC analysis shows that increased ICA is a very reliable test for TID predictions in children and adolescents, with an AUC of 0.761. However, the AUCs for MiR375 and GADA were 0.158 and 0.394, respectively.

Although GADA is a more potent marker for autoimmune damage to islet β cells. Our findings are in line with another study done among kids with positive islet autoantibodies, which showed a lesser significance for GADA in diagnosing T1D [28].

Swolin-Eide, et al.2023 [29]. identifies miRNAs as potential biomarkers for clinical use in T1D, and blood miRNA levels can be targeted for personalized therapy to decrease long-term problems associated with diabetes. In the development of type 1 diabetes, imbalanced miRNAs have been identified. By bringing these miRNAs back to their usual levels and employing miRNA mimics or inhibitors, it’s possible to enhance the production and release of insulin, as well as increase the sensitivity to insulin [30].

MiR375 measurement in diabetic individuals is now required to verify its value as a novel biomarker in the early stages of the disease. And to evaluate whether this miRNA represents simply metabolic alterations associated with T1D or a chronic disease that causes pancreatic inflammation and beta cell death.

Briefly, our study indicates that miR-375 is significantly downregulated in children with T1D and furtherly under-expressed in their siblings. In addition, miR-375 is significantly inversely correlated to HbA1c levels with a significant positive correlation to other biochemical parameters in children with T1D and their siblings. Our findings suggest that under expression of miR-375 plays a potential role in beta-cell function and glycemic control Further research and understanding of miR-375 mode of action is needed to investigate its possible use as an early predictive and progressive biomarker of T1D in addition to its utilization as a potential therapeutic target.

## A limitation of this study

It was a cross-sectional study, with a small sample size sample, which may reduce the statistical power of results. Therefore, subsequent work with a large sample size and multiple center studies is recommended.

Future research, a longitudinal study, and sharing of multiple centers. C Peptide will be added to investigations to evaluate beta cell function. Bioinformatics will be used in the evaluation of the role of miR-375 in T1D pathogenesis.

## Conclusion

“Our study indicates that miR-375 is significantly downregulated in children with T1D and their siblings, suggesting its potential role as a biomarker for beta-cell function and glycemic control.”

## Electronic Supplementary Material

Below is the link to the electronic supplementary material.

Supplementary Material 1

## Data Availability

The datasets used in this study are available in public databases as indicated in the paper citations. However, generated data and figures during this study are available on request to the corresponding author.
