# Supplementary material for: MiR-375: it could be a general biomarker of metabolic changes and inflammation in type 1 diabetes patients and their siblings
Source: J Endocrinol Invest. 2024 Oct 25;48(3):757–64. doi: 10.1007/s40618-024-02474-4 (PMC11876202; doi:10.1007/s40618-024-02474-4)
Supplement: Supplementary file 1 — Supplementary Material 1 [file 40618_2024_2474_MOESM1_ESM.docx]

**
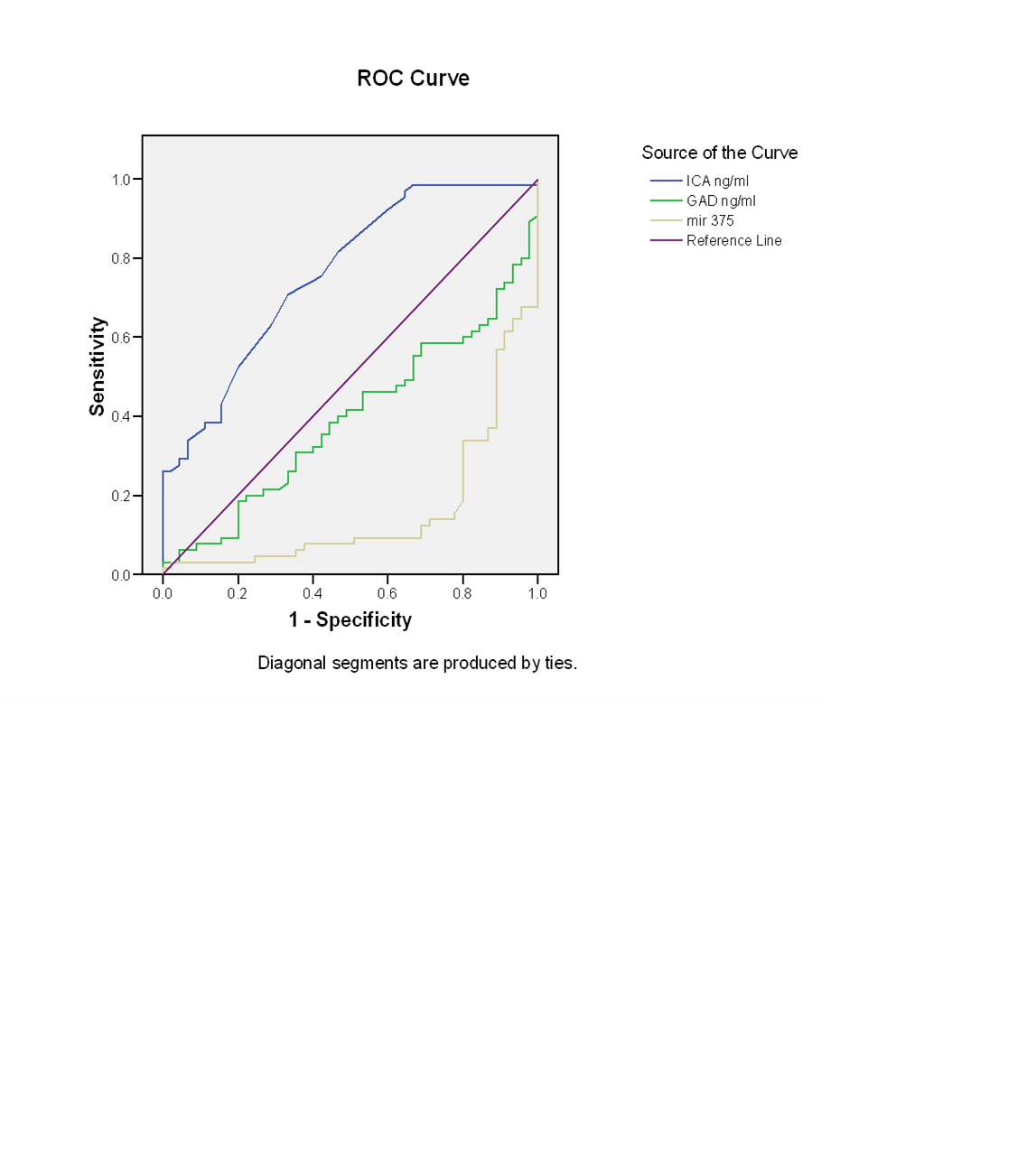
Fig 1: ROC curves for ICA islet cell, GADA glutamic acid decarboxylase autoantibodies and mir 375.** The diagonal line represents a test with no diagnostic value.
